# Plasma and aqueous levels of alarin and adipsin ın patients with and without diabetic retinopathy

**DOI:** 10.1186/s12886-022-02403-0

**Published:** 2022-04-18

**Authors:** Fatih Cem Gül, Sabiha Güngör Kobat, Fatih Çelik, Süleyman Aydin, Ramazan Fazıl Akkoç

**Affiliations:** 1 Medical Doctor Ophthalmology Clinic, Universal Eye Center, Elazig, Turkey; 2 Medical Doctor Department of Ophthalmology Clinic, Firat University, Elazig, Turkey; 3 Medical Doctor Ophthalmology Clinic, Elazig City Hospital, Elazig, Turkey; 4 Prof Dr Department of Biochemistry, Firat University, Elazig, Turkey; 5 Assistant professor Department of Anatomy, Firat University, Elazig, Turkey

**Keywords:** Diabetic retinopathy, Alarin, Adipsin, Plasma, Aqueous

## Abstract

**Backround:**

Diabetic retinopathy is a disease seen with microvascular complications as a result of hyperglycemia and insulin resistance. Alarin and Adipsin are molecules with a role in energy and glucose metabolism. The aim of this study was to determine plasma and aqueous levels of Alarin and Adipsin in patients with and without diabetic retinopathy to evaluate their potential roles in diabetic retinopathy.

**Methods:**

The study included one eye from each of 20 cataract patients without diabetes (C), 20 cataract patients with diabetes and without diabetic retinopathy (DM + C), and 20 cataract patients with diabetes and diabetic retinopathy (DR + C). Plasma and aqueous humour samples were taken from all patients during the cataract operation. Alarin and Adipsin levels were examined with the enzyme-linked immunosorbent assay (ELISA) method.

**Results:**

Both plasma and aqueous Alarin levels were significantly higher in the patients with diabetic retinopathy than in the control group (*p* < 0.001, *p* = 0.006). Adipsin levels were found to be significantly higher in plasma in the control group than in the DR + C group and significantly higher in aqueous in the DR + C group than in the control group (*p* < 0.001, *p* < 0.001).

**Conclusion:**

These findings suggest that Alarin and Adipsin may play important role in diabetic retinopathy.

## Backround

Diabetes mellitus (DM) is a chronic condition characterized by hyperglycemia and caused by insulin insufficiency or resistance. Diabetes and its complications are caused by a disruption in the physiological balance of the molecules that regulate glucose metabolism. Diabetic retinopathy is the most common complications of diabetes and the major cause of visual loss in working-age adults [1]. Diabetic microvascular complications are related to the severity and duration of hyperglycemia [2, 3]. The disease begins in a non-proliferative phase, progresses to a proliferative phase when uncontrolled diabetes is not treated, and results in significant eyesight loss.

Diabetic retinopathy is a classic example of microvasculopathy triggered by hyperglycemia.

Dysfunction, death and insufficient renewal in endothelial and vascular smooth muscle cells, capillary endothelial cells and pericytes induced by hyperglycemia have a role in the pathogenesis of diabetic microvasculopathy [4]. Insulin resistance has an important role in microvascular damage. Due to increased adipose tissue, the effects of insulin at the tissue level change as a result of the release of adipokines originating from visceral, cutaneous and perivascular adipose tissue at different levels. While insulin causes vasodilation via the PI3 kinase-mediated nitric oxide pathway under normal conditions, this balance shifts to the endothelin pathway due to adipokine-mediated insulin resistance, resulting in vasoconstriction. Microvascular damage has been shown to emerge as a result of this change associated with a decreased nitric oxide pathway and increased endothelin pathway [5]. It has been shown that defects in glucose uptake into tissues, insulin resistance, and retinopathy due to hyperglycemia develop as a result of disruptions in the metabolism of molecules such as leptin, resistin and adiponectin originating from adipose tissue [6].

Alarin, which is an adipokine thought to have an effect on glucose metabolism, is a cytokine containing 25 amino acids which is a member of the galanine peptide family isolated from gangliocytes of human neuroblastic tumours. It is named as a splice variant of the galanin-like peptide mRNA originating from N-terminal alanine and c-terminal serine [7]. Alarin was first determined in ganglionic cells, then around the ocular blood vessels which show vasoactive activity in blood flow regulation [8]. In addition, Alarin -like immunoreactivity has been determined in the human cornea, conjunctiva, iris, retinal vessels and internal choroidal plexus [9]. Previous animal studies have determined that peripheral and intraventricular administration of Alarin caused an increase in food intake, body weight, and insulin-mediated glucose intake of tissues [10].

Adipsin is another molecule thought to be effective in glucose metabolism. Adipsin, the first identified adipokine, is a member of the serine protease family and was first identified in 3T3 adipocytes [11]. In later studies Adipsin has been shown to be complement factor D, which participates in the alternative pathway of the complementary system [12]. Adipsin also has an effect on insulin secretion, which helps to maintain a stable blood glucose level. A remarkable reduction in plasma glucose levels was observed in rats treated with Adipsin, as well as an increase in fasting or glucose-induced insulin levels [13].

In our extensive literature review, we could not find any study showing the relationship of Alarin and Adipsin with diabetic retinopathy. And then again, as remembered disorder of *carbohydrate metabolism* is generally considered the primary culprit in the development and progression of *diabetic retinopathy*. So these proteins are a logical culprit to consider in *diabetic retinopathy*. Therefore the aim of this study was to compare the plasma and aqueous levels of Alarin and Adipsin, which were thought to have an effect on diabetic retinopathy, in patients with diabetic retinopathy, patients with diabetes but no retinopathy, and those with neither diabetes nor retinopathy, and to thereby be able to determine the potential role of these molecules in the etiopathogenesis of diabetic retinopathy.

## Methods

The study was performed in adherence with the tenets of the Declaration of Helsinki and was approved as a prospective study by the Ethics Committee of Firat University Faculty of Medicine (approval no: 2020/08–43).

The study included patients who presented at the Eye Diseases Polyclinic of Elazig Health Sciences University because of reduced vision, were diagnosed with cataract after a detailed ophthalmological examination, and underwent cataract surgery. Diabetes was diagnosed by an endocrine and metabolic disease physician according to the ADA guidelines [14], and a cataract and diabetic retinopathy diagnosis was established by ophthalmologists. The study included one eye from each of 20 cataract patients without diabetes (C), 20 cataract patients with diabetes and without diabetic retinopathy (DM + C), and 20 cataract patients with diabetes and nonproliferative diabetic retinopathy (DR + C). Patients with dot-blot hemorrhages, cotton-wool spots, venous beading, or intraretinal microvascular anomalies in the absence of neovascularization in the retina were evaluated as non-proliferative diabetic retinopathy and included in the study. Patients with proliferative diabetic retinopathy (neovascularization or vitreous hemorrahage) were excluded from the study. Cases with surgical complications (posterior capsule rupture, nucleus drop, zonular defect, etc.) or additional ophthalmological diseases (glaucoma, age-related macular degeneration, retinopathy, systemic arterial hypertension, otoimmune disease etc.) were excluded. To control the effects of certain parameters on our study, participants with similar DM duration, DM treatment (diet, exercise, metformin [2000 mg/day], and gliclazide (30 mg/day) or diet, exercise, metformin [2000 mg/day], and insülin [0.3–0.4 IU/kg/day]) were chosen.

### Collection of biological samples

After an 8–12-h fasting period, a 10 cc blood sample was taken from each patient in the morning into a tube containing aprotinin (BD Vacutainer SST II Advance, BD, Plymouth, UK).

In all patients, BMI (body mass index:kg/m^2^), fasting plasma glucose (FPG), HbA1c, and lipid profile (LDL, HDL, triglycerides) were examined. The collection, storage, and preservation of these samples were previously described [15]. The obtained blood samples were centrifuged at 4000 rpm for 10 min, and the plasma obtained was placed in small volume tubes and stored at -80 °C until assay of Alarin and Adipsin. All the patients in the study group were applied with phacoemulsification + intraocular lens implantation. During the cataract operation, aqueous samples were taken and stored at -80 °C until assay.

### Surgical Method

Phacoemulsification was used throughout this study as described previously [16]. Thirty minutes before the operation, alprazolam (0.5 mg) was orally administered for sedation of the patient. Topical cyclopentolate (1%), tropicamide (0.05%), and phenylephrine (2.5%) were used for pupil dilation. For local anesthesia, topical % 0.5 Propakain HCL dropped on corneal and conjunctival surface. The cornea was incised at the 9 o’clock positions with a 20-G MVR knife and aqueous samples taken from this incision from the anterior chamber. In addition another corneal incision was made at 1 o’clock. Viscoelastic material was inserted. At the 11 o’clock position, a corneal incision was made using a number 3 knife. The lens was emulsified with a stable salt solution (BSS), followed by the horizontal chop method with hydrodissection and hydrodelineation. The remaining lens material was removed by manual irrigation and aspiration (I/A) of the cannula. A foldable intraocular lens was installed using a cartridge system. The viscoelastic material inserted into the anterior chamber was removed using the manual I/A method. The incision site was closed with stromal hydration, and any wound leakage was controlled.

### Biochemical Analyses of Biological Samples

Plasma Alarin and Adipsin levels were examined using the Human Alarin, Adipsin ELISA Kit (Sunred Biological Technology, Shanghai, China) in a plate-washing -incubation CombiWash device (Human Diagnostics, Wiesbaden, Germany) in accordance with the study procedures determined in the kit catalogue, and the absorbance measurement was taken with a Chromate 4300 Microplate Reader (Awareness Technology, Palm City, USA).

Aqueous analyzes were performed according to a previously published methods [15]. Two Aqueous liquids and blood samples were enriched with increasing amounts of Adipsin or Alarin. The percentage recovery was calculated as follows: recovered value/expected value × 100.

The measurement range human alarin kit was 5 to 1500 pg/mL and the sensitivity was determined by the manufacturer at 4.638 pg/mL. The intra-assay and inter-assay coefficients of variation for alarin were < 10% and < 12%, respectively. The measurement range of the human Adipsin kit 0.5 to 100 ng/mL and the sensitivity was the determined by the manufacturer at 0.472 ng/mL. The intra-assay and inter-assay coefficients of variation for Alarin were < 10% and < 12%, respectively.

### Assay validation of kits for aqueous fluids in our laboratory

Aqueous assay validation was performed according to a previously published method by Aydin [15], as was briefly described below.

**Linearity:** Two aqueous liquids and blood samples were diluted (1/2, 1/4, 1/8) with distilled water in order to find the Alarin and Adipsin linearity.

**Recovery:** Two Aqueous liquids and blood samples were enriched with pure amounts of Alarin and Adipsin. The percentage recovery was calculated as follows: recovered value/expected value × 100.

**The *coefficient of variation* (CV):** The intra-assay (within-day) and inter-assay variation (between days) were determined for two different two aqueous liquids and blood samples using the means of 2–3 replicates of each. The coefficient of variation (CV) is calculated as: CV = Standard Deviation (SD)/Mean concentration.

## Statistical Analysis

Data obtained in the study were analysed statistically using the Statistical Package for the Social Sciences (SPSS) version 22.0 software (SPSS Inc., Chicago, IL, USA). To determine the signifiance of the difference between the groups in respect of age, gender, FPG, HbA1c, lipid profile, plasma and aqueous alarin and adipsin levels, the Mann Whitney U-test was applied. A value of *p* < 0.05 was accepted as statistically significant. To make the difference more clear, logistic regression was used. Plasma adipsin and plasma alarin levels were found to be significant in separating the groups (*p* < 0.001, *p* = 0.014, respectively). According to the variables of adipsin plasma, adipsin aqueous, alarin plasma and alarin aqueous in the nominal logistic regression model's classification model, the model correctly classified the C, DM + C, and DR + C groups at an 80% (Tables 1 and 2). In addition, ROC analysis was applied. Plasma adipsin levels were found to be significant in differentiating the first group from the other groups (*p* < 0.001). Aqueous adipsin, plasma alarin and aqueous alarin levels were found to be significant in differentiating the third group from the other groups, respectively (*p* < 0.001, *p* < 0.001, *p* = 011).

**Table 1:** Logistic regression model for adipsin plasma, adipsin aqueous, alarin plasma and alarin aqueous. (C: Cataract; DM: Diabetes mellitus; DR: Diabetic retinopathy)

**Parameter Estimates**									
group^a^		B	Std. Error	Wald	df	Sig	Exp(B)	95% Confidence Interval for Exp(B)	
								Lower Bound	Upper Bound
DM + C	Intercept	,102	2,954	,001	1	,972			
	Adipsinplasma	-,105	,045	5,567	1	,018	,900	,825	,982
	Adipsinaqueous	,059	,049	1,418	1	,234	1,061	,963	1,169
	Alarinplasma	,033	,017	3,894	1	,048	1,033	1,000	1,067
	Alarinaqueous	,012	,030	,169	1	,681	1,012	,955	1,074
DR + C	Intercept	-2,120	3,859	,302	1	,583			
	Adipsinplasma	-,194	,068	8,171	1	,004	,824	,722	,941
	Adipsinaqueous	,114	,066	3,000	1	,083	1,121	,985	1,275
	Alarinplasma	,047	,019	6,193	1	,013	1,048	1,010	1,088
	Alarinaqueous	,030	,033	,862	1	,353	1,031	,967	1,099

a. The reference category is: C

**Table 2:** Classification table for logistic regression model (C: Cataract; DM: Diabetes mellitus; DR: Diabetic retinopathy)

Classification				
Observed	Predicted			
	C	DM + C	DR + C	Percent Correct
C	20	0	0	100,0%
DM + C	4	12	4	60,0%
DR + C	0	4	16	80,0%
Overall Percentage	40,0%	26,7%	33,3%	80,0%

## Results

The validation of the kits we use has been made in our laboratory. Results of the linearity of used kits in biological samples were summarized in Table 3. Table 4 indicated recovery assay results of kits used through this study.. Intra assay values were calculated as 7.8% and 7.1% for Adipsin and Alarin in our laboratory, while inter assay values were recorded as 11.4% and 14.6% for Adipsin and Alarin, respectively, in our laboratory (Table 3, Table 4).

**Table 3:** Linearity of Kits in biological samples used through this study (Adipsin concentrations in ng/mL, Alarin in pg/mL)

		**Undiluted (100%)**	**1/2**	**1/4**	**1/8**
Adipsin	Aqueous-1	93.18	87.1 (93.4%)	92.4(98%)	89.8 (96.3%)
	Aqueous-2	100.98	104.1(103%)	106.8 (105.7%)	98.4 (97.4%)
	Blood-1	60.23	67.2 (111%)	58.4 (96.9%)	60.2 (99.9%)
	Blood-2	63.54	57.4 (90.3%)	64.2 (101%)	64.8 (102%)
Alarin	Aqueous-1	178.92	184.8(103%)	188.2(105%)	172.4 (96.3%)
	Aqueous-2	181.37	176.2 (97%)	178.4 (98.3%)	196.8 (108.5%)
	Blood-1	93.18	102.2 (109%)	96.2 (103%)	88.8 (95.2%)
	Blood-2	109.80	98.4 (89.6%)	108.4 (98.7%)	112.8 (102%)

**Table 4:** Recovery Assay (RCA) of Kits in biological samples used through this study. (Adipsin concentrations in ng/mL, Alarin in pg/mL)

	**Samples**	**Initial concentration**	**Added**	**Recovered**	**Expected**	**Recovery (%)**
Adipsin	Aqueous-1	106.83	64	184.2	170.83	107.1
	Aqueous-2	133.10	64	209.4	197.10	106.2
	Blood-1	56.88	64	119.8	120.88	99.1
	Blood-2	59.42	64	126.8	123.42	102.8
Alarin	Aqueous-1	181.37	400	613.4	581.37	105.5
	Aqueous-2	171.57	400	581.8	571.57	100.7
	Blood-1	80.68	400	478.6	480.68	99.5
	Blood-2	89.77	400	494.8	489.77	101.0

The 20 cataract patients without diabetes or retinopathy (C) comprised 12 (60%) males and 8 (40%) females with a mean age of 65.95 ± 2.85 years. The 20 cataract patients with diabetes and without diabetic retinopathy (DM + C) comprised 7 (35%) males and 13 (65%) females with a mean age of 65.10 ± 1.83 years. The 20 cataract patients with diabetes and diabetic retinopathy (DR + C) comprised 10 (50%) males and 10 (50%) females with a mean age of 64.85 ± 1.66 years. No statistically significant difference was determined between the groups in respect of age (*p* > 0.05 for all) (Table 5).

**Table 5:** Demographic characteristics of the patients

	C	DM + C	DR + C
Age (years)	65.95 ± 2.85	65.10 ± 1.83	64.85 ± 1.66
BMI (kg/m^2^)	26.07 ± 2.26	32.76 ± 1.68*	34.76 ± 2.66**
FPG (mg/dL)	91.65 ± 7.25	155.95 ± 20.73*	166.80 ± 15.37*
HbA1c (%)	5.53 ± 0.14	7.06 ± 0.30*	8.32 ± 0.40**
HDL (mg/dL)	47.68 ± 2.38	40.49 ± 1.28*	39.88 ± 1.57*
LDL (mg/dL)	127.81 ± 6.45	156.09 ± 6.49*	159.81 ± 5.07*
Triglyceride (mg/dL)	138.10 ± 23.34	177.04 ± 5.64*	188.92 ± 5.87**

*C* Cataract; *DM* Diabetes mellitus; *DR* Diabetic retinopathy; *BMI* Body mass index; *FPG* Fasting plasma glucose; *HbA1c* hemoglobinA1c, *HDL* High density lipoprotein; *LDL *Low density lipoprotein, *: compared with Group C *p* < 0.05 (Mann Whitney *U*), **: compared with Group C and Group DM + C *p* < 0.05 (Mann Whitney *U*)

The FPG levels in groups C, DM + C, and DR + C were determined as 91.65 ± 7.25 mg/ dL, 155.95 ± 20.73 mg/dL, and 166.80 ± 15.37 mg/dL, respectively. The FPG levels in group DR + C and group DM + C were determined to be statistically significantly higher than in group C (*p* < 0.001, *p* < 0.001). No statistically significant difference was determined between groups DR + C and DM + C (*p* > 0.05). The HbA1c levels in groups C, DM + C and DR + C were determined as 5.53 ± 0.14, 7.06 ± 0.30, and 8.32 ± 0.04, respectively. The HbA1c levels in groups DR + C and DM + C were determined to be statistically significantly higher than in group C (*p* < 0.001, *p* < 0.001). A statistically significant difference was determined between the HbA1c levels of groups DR + C and DM + C (*p* < 0.001). The BMI values in groups C, DM + C and DR + C were determined as 26.07 ± 2.26, 32.76 ± 1.68, and 34.76 ± 2.66 respectively. The BMI levels in groups DR + C and DM + C were determined to be statistically significantly higher than in group C (*p* < 0.001, *p* < 0.001). A statistically significant difference was determined between the BMI values of groups DR + C and DM + C (*p* = 0.007) (Table 5).

The HDL levels of groups C, DM + C and DR + C were determined as 47.68 ± 2.38 mg/dL, 40.49 ± 1.28 mg/dL, and 39.88 ± 1.57 mg/dL, respectively. The HDL levels in groups DM + C and DR + C were significantly lower than those in group C (*p* < 0.001, *p* < 0.001). No statistically significant difference was determined between the HDL levels of groups DR + C and DM + C (*p* > 0.05). The LDL levels of groups C, DM + C and DR + C were determined as 127.81 ± 6.45 mg/dL, 156.09 ± 6.49 mg/dL L, and 159.81 ± 5.07 mg/dL, respectively. The LDL levels in groups DM + C and DR + C were significantly higher than those in group C (*p* < 0.001, *p* < 0.001). No statistically significant difference was determined between the LDL levels of groups DR + C and DM + C (*p* > 0.05). The triglyceride (TG) levels of groups C, DM + C and DR + C were determined as 169.97 ± 5.52 mg/dL, 177.04 ± 5.64 mg/dL, and 188.92 ± 5.87 mg/dL, respectively. The TG levels in groups DM + C and DR + C were significantly higher than those in group C (*p* < 0.001, *p* < 0.001). A statistically significant difference was determined between the TG levels of groups DR + C and DM + C (*p* < 0.001) (Table 5).

**Fig. 1:**
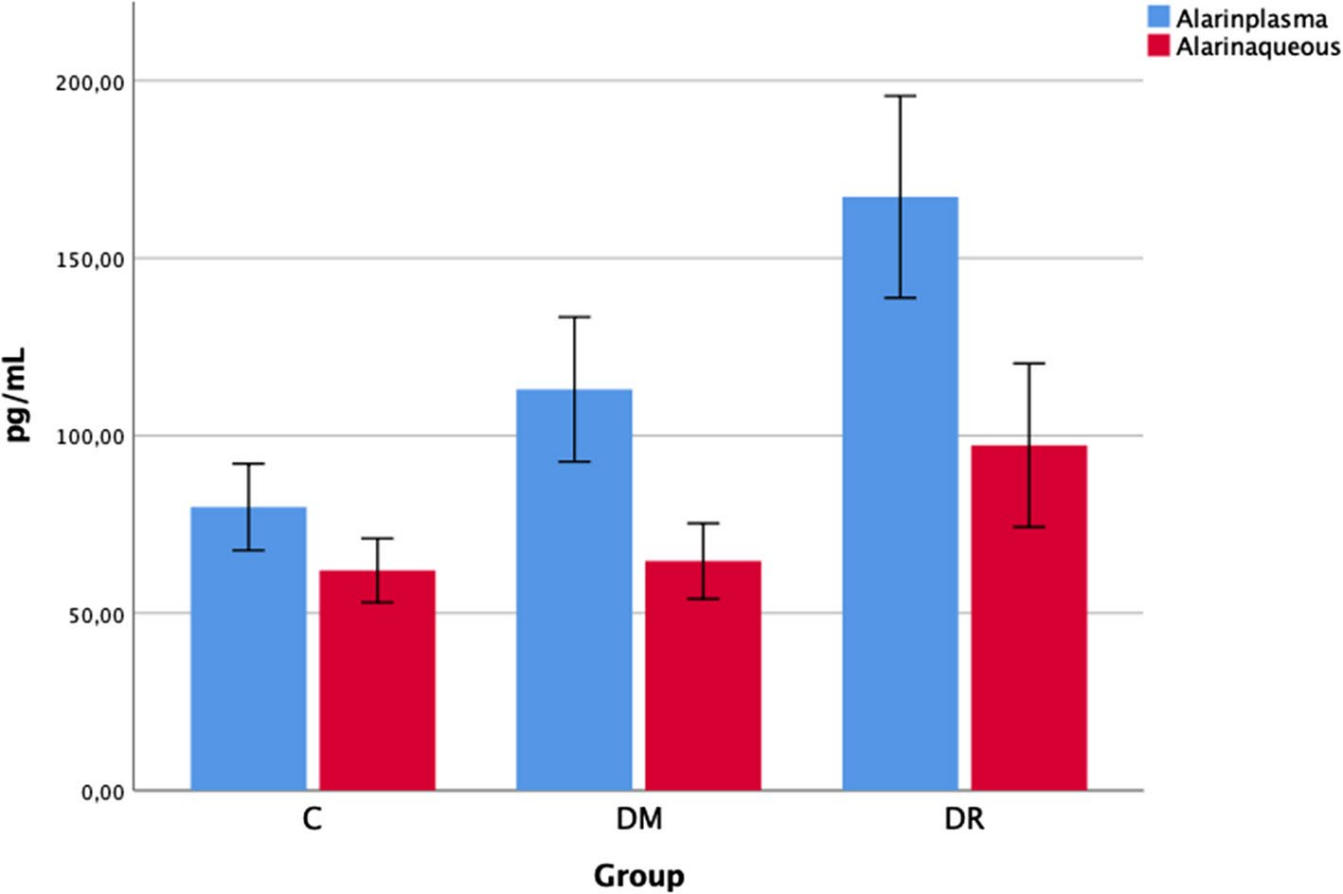
Alarin levels in the aqueous humor and blood of patients with DR + C, DM + C and C

The plasma Alarin levels were determined as 79.56 ± 26.09 pg/mL in group C, 113.01 ± 43.52 pg/mL in group DM + C, and 167.23 ± 60.77 pg/mL in group DR + C. The plasma Alarin levels in both groups DM + C and DR + C were statistically significantly higher than those in group C (*p* = 0.006, *p* < 0.001). The plasma Alarin levels in the DR + C group were statistically significantly higher than those of the DM + C group (*p* = 0.003) (Fig. 1).

The aqueous Alarin levels were determined as 61.99 ± 19.27 pg/mL in group C, 64.64 ± 22.76 pg/mL in group DM + C, and 97.26 ± 49.20 pg/mL in group DR + C. The aqueous Alarin levels in group DR + C were statistically significantly higher than those in group C and group DM + C (*p* = 0.006, *p* = 0.011). No statistically significant difference was determined between the aqueous Alarin levels of group C and group DM + C (*p* > 0.05) (Fig. 1).

The plasma Adipsin levels were determined as 66.21 ± 19.86 ng/mL in group C, 41.60 ± 13.35 ng/mL in group DM + C, and 30.90 ± 10 ng/mL in group DR + C. The plasma Adipsin levels in group C were statistically significantly higher than those in both groups DM + C and DR + C (p < 0.001, *p* < 0.001). The difference between the plasma Adipsin levels in group DM + C and group DR + C was determined to be statistically significant (*p* = 0.007).

**Fig. 2:**
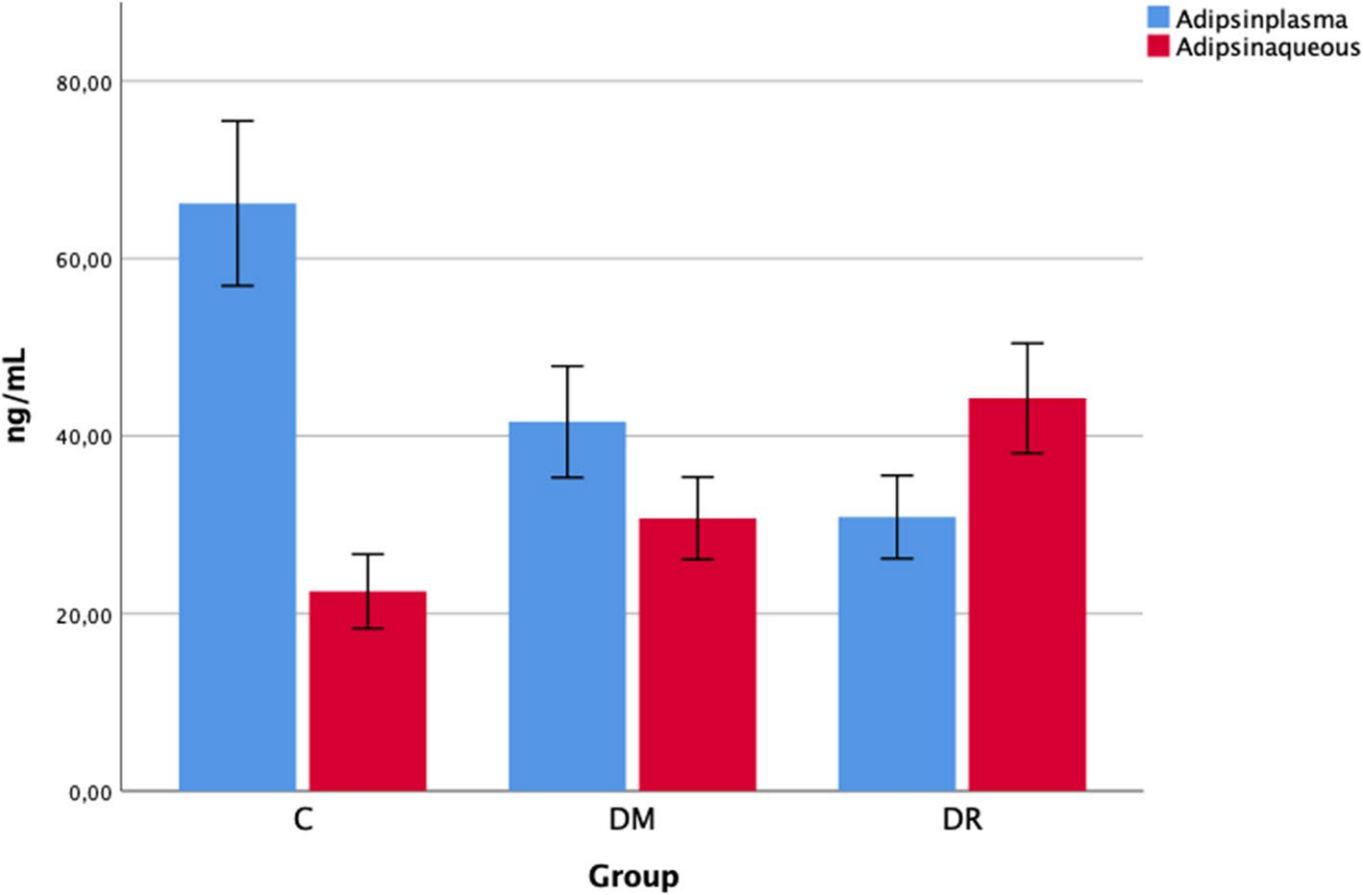
Adipsin levels in the aqueous humor and blood of patients with DR + C, DM + C and C

The aqueous Adipsin levels were determined as 22.52 ± 8.94 ng/mL in group C, 30.75 ± 9.91 ng/mL in group DM + C, and 44.24 ± 13.24 ng/mL in group DR + C. The aqueous Adipsin levels in both groups DM + C and DR + C were statistically significantly higher than those in group C (*p* = 0.009, *p* < 0.001). The aqueous Adipsin levels in group DR + C were determined to be statistically significantly higher than those in group DM + C (*p* = 0.001) (Fig. 2).

**Fig. 3:**
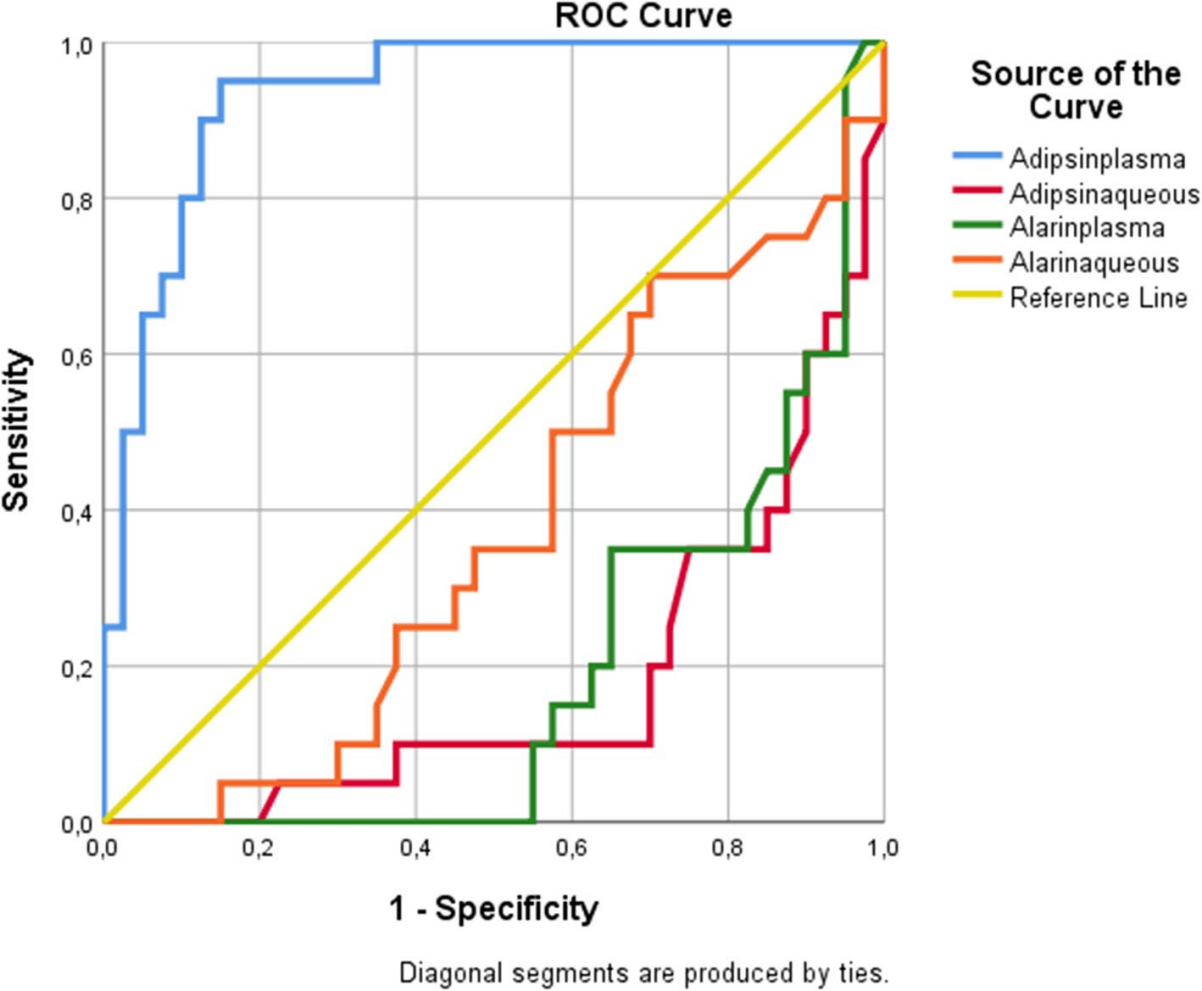
Roc curve of C for plasma adipsin, aqueous adipsin, plasma alarin, aqueous alarin

**Fig. 4:**
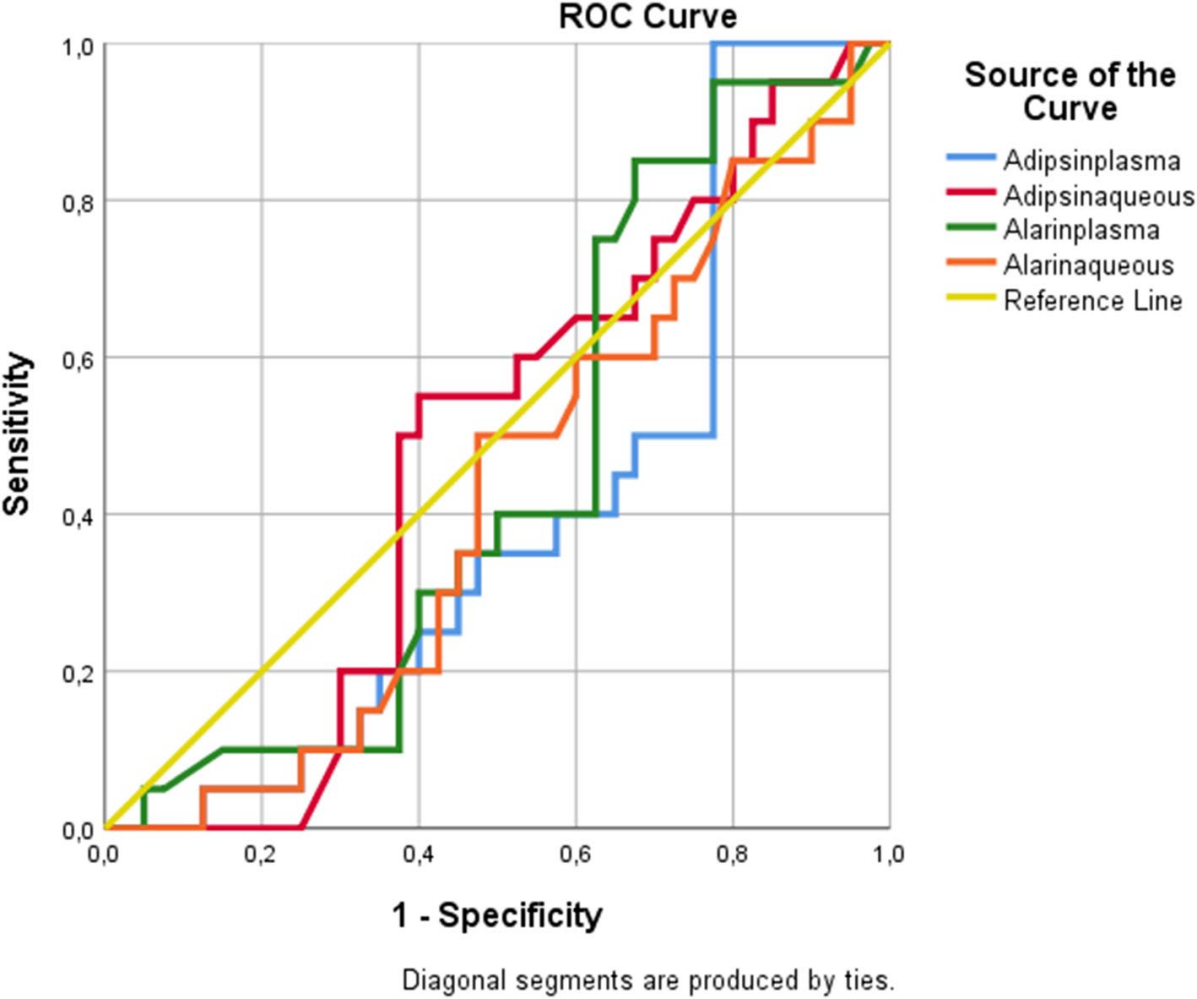
Roc curve of DM + C for plasma adipsin, aqueous adipsin, plasma alarin, aqueous alarin

**Fig. 5:**
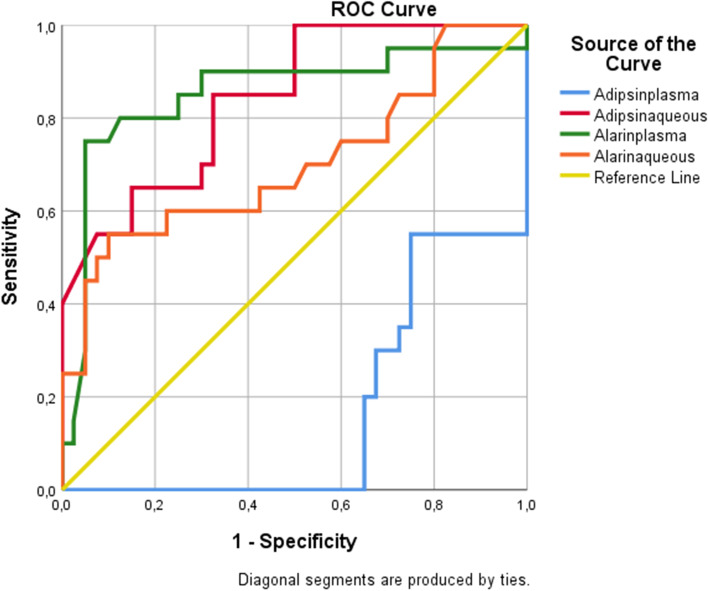
Roc curve of DR + C for plasma adipsin, aqueous adipsin, plasma alarin, aqueous alarin

In addition, ROC curve of C, DM + C and DR + C for plasma adipsin, aqueous adipsin, plasma alarin, aqueous alarin are given in the Figs. 3, 4 and 5 respectively.

## Discussion

The results of this study showed that the fasting plasma glucose levels, HbA1c and BMI values were significantly higher in patients with DR + C than in the DM + C and C groups. When the Alarin results were examined, the plasma Alarin levels were seen to be significantly higher in the DM + C and DR + C groups compared to the C group (*p* = 0.006, *p* < 0.001). In vivo and in vitro animal studies have shown that Alarin reduced insulin resistance and lowered increased insulin and glucose levels [17, 18].

In vivo studies have also reported that Alarin may be released in response to metabolic stress factors such as abdominal obesity, insulin resistance, dyslipidemia, hyperglycemia, and hypertension. [19]. In patients with metabolic syndrome and obesity, Alarin levels have been determined to be higher than those of control groups. There has been observed to be a compensatory elevation in Alarin levels in response to oral glucose intake. It has been emphasized that high Alarin levels may be related to resistance to Alarin with a mechanism similar to insulin resistance. [19]. The patients in our study consisted of patients with a body mass index of 30 and above, with metabolic syndrome-like findings.. The elevated Alarin levels determined in the DM + C and DR + C of the current study could be associated with both a compensatory response to chronic hyperglycemia and with potential Alarin resistance.

In mice, Alarin injection into the central nervous system or peripheral administration causes orexigenic behavior and weight gain, as well as a decrease in plasma insulin and glucose levels [17, 18]. Reduced glucose intake to skeletal muscle is the key factor in insulin resistance. Both central and peripheral administration of Alarin has been shown to increase the passage of glucose to tissues, especially skeletal muscle, by the GLUT 4 pathway [17]. In addition, the effects of Alarin on insulin sensitivity have been found to emerge by reducing RBP4 and increasing adiponectin levels [18]. Alarin’s orixogenic effects are thought to be NPY-mediated. However, no Alarin-specific receptor revealing the central effects of Alarin has been determined [18]. It is known that chronic hyperglycemia can cause diabetic retinopathy through several mechanisms. The elevated Alarin levels in the current study may be associated with chronic hyperglycemia. However, high Alarin levels not at a level to prevent hyperglycemia, may be related to resistance to Alarin as stated in previous studies, predominance of the orexigenic effect or as yet unrevealed specific Alarin receptor-mediated complex mechanisms. Therefore, although the Alarin levels were high in current study, they were not at an effective level to prevent the development of diabetic retinopathy. Further studies of Alarin-specific receptors and effect mechanisms could reveal the potential for Alarin to be used especially in hyperglycemia treatment.

Alarin immunoreactivity has been determined in human ocular epithelial cells (cornea, conjunctiva, ciliary body), ocular blood vessels (iris, retina, choroid) and neurons (retina and choroid) [9]. It has been reported that Alarin may function as a neuropeptide in interaction with other neurotransmitters and neuropeptides, which probably have neuromodulator functions in the choroid and retina. [9]. Alarin, which has been found in the iris blood vessels and ciliary body, is considered to have a role in the immune defense of the eye, as well as maintaining the function of the corneal endothelium in the anterior chamber, with neurotransmitter and neuropeptide-like actions [9]. The activity of alarin detected around retinal and choroidal vessels suggests that it may be important in ocular blood flow hemostasis due to the change in blood vessel diameter. Alarin shows a dose-related vasoconstriction, anti-edema and anti-inflammatory effect following subcutaneous injection in the skin and and this effect supports its potential effects on retinal vessels [8]. In the current study, the Alarin levels in the aqueous humour were determined to be significantly high in the DR + C group compared to the DM + C and C groups (*p* = 0.011, *p* = 0.006). High levels of Alarin in aqueous humor in the current study suggest that Alarin may play a compensatory role in diabetic retinopathy, which is associated with changes in retinal microvascular structure, macular edema, and inflammation. Future studies with Alarin, we believe, will be important in determining whether the vasoconstrictor, anti-edema, and anti-inflammatory effects of this molecule can be used to treat diabetic retinopathy.

When the Adipsin results in the current study were examined, the Adipsin levels in the DR + C group and DM + C group were determined to be statistically significantly lower than those of the C group (*p* < 0.001, *p* < 0.001). Adipsin is an important molecule, expressed from adipose tissue, which has an effect on glucose and lipid metabolism. Adipsin provides complement factor mediated insulin secretion from pancreatic beta cells, transfer of glucose from plasma to tissue and storage by converting to triglycerides in tissue [20]. In mice with defective Adipsin gene, Lo et al. determined reduced glucose tolerance and insufficient insulin secretion. As a result of treatment of diabetic db/db mice with vectors expressing Adipsin, there was seen to be an improvement in glucose tolerance and a reduction in FPG. At the same time, fasting and glucose-induced insulin levels increased. Even if beta cell function was insufficient, the Adipsin levels were determined to be low [13]. Adipsin levels have been determined to be significantly low in db/db and ob/ob mice with high glucose levels and a decrease in Adipsin levels has been determined in a hyperinsulinemic and hyperglycemic environment created with continuous glucose infusion [21, 22]. In a study by Banoy et al., it was emphasised that high Adipsin levels reduced the risk of developing diabetes in middle-aged adults [23]. When hyperglycemia and impaired insulin expression causing diabetic retinopathy are considered, the low Adipsin levels are consistent with literature and are an expected finding. Therefore, we believe Adipsin could be a potential treatment option for reducing the severity of diabetic retinopathy and its complications.

There are several conflicting results in literature related to Adipsin levels in obese patients. BMI values have been reported to be ≥ 35 in obese patients determined with high Adipsin levels and this elevation has not been determined in those with a lower BMI [20]. In a study by Wang et al., Adipsin levels were determined to be low and insulin resistance was high in patients with BMI ≥ 25 [24]. It has also been suggested that this elevation could be due to subcutaneous adipose tissue in patients with high Adipsin levels, and Adipsin levels have not been found to be high in those with visceral adipose tissue [23]. In addition, it has been reported that as a result of persistent glucose elevations in type 2 diabetes patients in particular, compensation in adipose tissue is impaired and Adipsin levels could decrease [13]. Besides the reasons stated above, these differences can be affected by many factors such as the duration of diabetes, racial differences, patient selection criteria, and method of determination [25]. The patients included in the current study were obese with mean BMI of 32.76 ± 1.68 in the DM + C group and 34.76 ± 2.66 in the DR + C group. The Adipsin levels were determined to be low in the obese patients in the current study. It suggests that there may be a defect in the compensatory release of Adipsin from the adipose tissue despite the increased glucose levels to cause diabetic retinopathy, and that pancreatic beta cell function may be low in our patient group. The improvement in the metabolism of Adipsin as a result of the decrease in the BMI may constitute an important factor in the treatment of diabetes and its complications.

Adipokine receptors have been determined in the choroid, iris, ciliary body, and cornea [26]. Adiponectin treatment of laser-induced choroidal neovascularization in rats resulted in a reduction in vascular endothelial growth factor levels and a regression in neovascularization. [27]. Ricker et al. compared cases of recurrent retinal detachment due to proliferative vitreoretinopathy to cases of rhegmatogenous retinal detachment in their study, and reported no significant differences in Adipsin levels in subretinal fluid in both groups [28]. These studies demonstrate that Adipsin could have a role in physiological and pathological processes in the eye. In the current study, the Adipsin levels in the aqueous humour samples taken from the anterior chamber were determined to be significantly high in the DR + C and the DM + C compared to the C group. The aqueous Adipsin levels were also determined to be higher than in plasma. When it is considered that adiponectins have a role in wound healing and inflammatory response, Adipsin could have a role in the pathogenesis of diabetic retinopathy or in the local response to diabetic retinopathy. However, there is a need for further studies to determine whether or not Adipsin can be used locally in the eye in the treatment of diabetic retinopathy.

There were some limitations to the current study, primarily the relatively small number of patients and the cross-sectional design of the study.

## Conclusion

Alarin and Adipsin, which play an important role in the pathophysiology of diabetes and obesity, and have a regulatory role in hyperglycemia and insulin resistance, can be considered for use in the treatment of diabetes and associated complications. These findings suggest that the measurement of Alarin and Adipsin levels may support clinicians in determining the risk of diabetic retinopathy development. Nevertheless, there is a need for further more extensive studies to evaluate the role of Alarin and Adipsin in the treatment of diabetic retinopathy.

## Data Availability

The datasets used and/or analysed during the current study are available from the corresponding author on reasonable request.
